# Use of Foaming Disinfectants and Cleaners to Reduce Aerobic Bacteria and Salmonella on Poultry Transport Coops

**DOI:** 10.3390/ani8110195

**Published:** 2018-11-02

**Authors:** Carolee Hinojosa, David Caldwell, James Byrd, Robert Droleskey, Jason Lee, Phil Stayer, Erin Resendiz, Javier Garcia, Stephanie Klein, Denise Caldwell, Megan Pineda, Morgan Farnell

**Affiliations:** 1 Poultry Science Department, Texas A&M AgriLife Research & Extension, Texas A&M System, College Station, TX 77843, USA; caroleehinojosa@gmail.com (C.H.); caldwell@tamu.edu (D.C.); jtlee@tamu.edu (J.L.); erinjfowlkes@gmail.com (E.R.); garcia07@vt.edu (J.G.); sklein@tamu.edu (S.K.); meganpineda@tamu.edu (M.P.); 2 USDA, Agricultural Research Service, SPARC, College Station, TX 77840, USA; byrdmen8@yahoo.com (J.B.); bob.droleskey@ars.usda.gov (R.D.); denise.caldwell@ars.usda.gov (D.C.); 3 Sanderson Farms, Laurel, MS 39443, USA; phil.stayer@sandersonfarms.com

**Keywords:** cleaning and disinfection, biosecurity, food safety, transportation coops, poultry

## Abstract

**Simple Summary:**

Chicken coops are rarely washed and can soil poultry carcasses with fecal bacteria that may make people sick. Our laboratory applied two commercially available products to experimentally contaminated coops. One product contained bleach, potassium hydroxide and a foaming agent. The other product contained vinegar and hydrogen peroxide and was mixed with a detergent. Both products were applied using a firefighting apparatus known as a compressed air foam system (CAFS). These materials were washed away using a garden hose or pressure washer as the treatments called for. Surface swabs were collected prior to and after each treatment to determine the reduction of bacteria on the surface, which would be an indicator of sanitation. We found that both treatments significantly made the surface cleaner when compared to water alone. The application of these products via a CAFS may be a practical and expedient way to clean and disinfect poultry cages.

**Abstract:**

Transport coops are infrequently washed and have been demonstrated to cross-contaminate broiler carcasses. We hypothesized that peracetic acid or a chlorinated cleaner, commonly used within poultry processing plants, can also be used to disinfect transport coops when applied via a compressed air foam system (CAFS). A mixture of fresh layer manure and concentrated *Salmonella* Typhimurium (ST) was evenly applied to the floors of four pre-cleaned transport coops and allowed to dry for thirty minutes. Treatments consisted of a (1) water rinse only, (2) product application with a water rinse, (3) product application followed by power washing and (4) power washing followed by application of product. Each foaming treatment was applied with a compressed air foam system and allowed 10 min of contact time. Samples were aseptically collected from the transport coops prior to and following treatment using a sterile 2 × 2-inch stainless steel template and a gauze swab pre-enriched with buffered peptone water. The chlorinated cleaner significantly (*p* < 0.05) reduced aerobic bacteria and ST by 3.18 to 4.84 logs across application methods. The peroxyacetic acid (PAA) disinfectant significantly (*p* < 0.05) reduced aerobic bacteria and ST by 3.99 to 5.17 logs across application methods. These data indicate that a compressed air foam system may be used in combination with a commercially available cleaner or disinfectant to reduce aerobic bacteria and ST on the surfaces of commercial poultry transport coops.

## 1. Introduction

Transportation coops have been shown to be a vector for cross-contamination during the 3–12 h transportation and holding period that occurs before birds are processed [1]. These coops contain organic matter and microorganisms left by previously transported flocks [2]. Salmonella and *Campylobacter* levels can increase by 20 to 40% during loading, transportation, and holding before being processed [3,4,5]. Transportation is a known stress factor in poultry production and is why studies show increasing levels of microorganisms during this event [6]. Poultry transportation coops are not required to be cleaned and disinfected prior to reuse, which may lead to cross-contamination between broiler flocks [5,7]. Broilers determined to be negative for *Campylobacter* become positive post-transportation in coops previously used for transport of *Campylobacter* positive flocks [7]. Research has been conducted to evaluate reductions in bacteria present on transportation coops by washing and allowing an extended drying time. These methods were found to be successful, but were considered impractical for the industry since this would require more coops and a large amount of space for drying [3].

*Campylobacter* and Salmonella are a concern within the industry because of their prevalence in poultry products [8]. Disinfectants such as peroxyacetic acid (PAA) are currently used in chillers at poultry processing plants because of its ability to reduce microorganisms, such as *Campylobacter* and Salmonella [9]. Guidelines to control and prevent these two microorganisms have been written and are in place for the poultry industry [10]. Researchers have collected carcass samples within poultry processing plants to determine where the highest loads of *Campylobacter* were found [11]. Mechanical feather removal within the processing plant is one area where bacterial load has been shown to increase, picker fingers cross-contaminate feather follicles with high levels of organic matter containing microorganisms which may further contaminate carcasses [12]. Lowering the number of microorganisms and organic matter entering the plant from transportation coops should result in less organic matter on carcasses and possibility reducing cross-contamination.

The poultry industry may use firefighting foam to depopulate birds during a reportable disease outbreak. The emergency technique has been conditionally approved by the American Veterinary Medical Association and the USDA-Animal Plant Health Inspection Service [13]. Foam is a quick alternative method to depopulate broilers that can be less labor intensive than gas asphyxiation [14]. Using a compressed air foam system (CAFS) may also be an efficient way to disinfect and sanitize poultry transportation coops. Disinfecting treatments using CAFS have been shown to reduce aerobic bacteria on layer cages and broiler transportation coops [15,16]. The food industry uses foaming disinfectants and cleaners to reduce microbial surface contamination, suggesting that a scalable approach using CAFS has potential.

In this study, we evaluated peracetic acid and a foaming cleaner that is commonly used by the poultry industry. Peracetic acid is a mixture of hydrogen peroxide and acetic acid. It is a robust disinfectant that can tolerate high organic loads yet decomposes into relatively safe by-products. It denatures proteins and increases cell wall permeability. The foaming cleaner was a proprietary formulation consisting of 5–10% potassium hydroxide, 1–3% sodium hypochlorite and a foaming agent. Alkaline ingredients are used to saponify lipids and help with the removal of organic matter. While the foaming cleaner was not labeled as a disinfectant, the strong base and sodium hypochlorite were expected to have some antimicrobial activity. Chlorine products are inexpensive and effective disinfectants that can kill or damage microbes due to oxidation of proteins and disruption of cell membranes. Unfortunately, they are also quickly depleted in the presence of organic matter [17].

The objective of the current study was to evaluate the disinfection of poultry transportation coops using a foam cleaner (FC), PAA + FA, or PAA + FA with a high-pressure water rinse (HPWR) prior to or following the foam application on aerobic bacteria and *Salmonella* recovery. A field study was conducted at a commercial poultry processing facility. This trial evaluated PAA + FA alone and with a HPWR prior to the foam application to evaluate aerobic bacteria present on poultry transportation coops. We hypothesized that the application of disinfectants or cleaners with foam using the CAFS would significantly reduce *Salmonella* and aerobic bacteria on broiler transport coops.

## 2. Materials and Methods

### 2.1. Experimental Design

Lab Trial 1–Peroxyacetic Acid with a High-Pressure Water Rinse - Aerobes and Salmonella Recovery. Lab trial 1 used four transportation coops, with each one representing a different treatment. Treatments consisted of a: (1) low-pressure water rinse (LPWR) only; (2) PAA + FA; (3) HPWR followed by the PAA + FA; and (4) PAA + FA followed by a HPWR.

Lab Trial 2–Foam Cleaner with a High-pressure Water Rinse–Aerobes and Salmonella Recovery. Lab trial 2 used four transportation coops, with each one representing a different treatment. Treatments consisted of: (1) LPWR; (2) FC; (3) HPWR followed by the FC; and (4) FC followed by a HPWR.

Field Trial—Peroxyacetic Acid with a High-Pressure Water Rinse–Aerobes. The field trial was conducted at a broiler processing facility and used three transportation coops. Treatments consisted of: (1) LPWR; (2) PAA + FA; and (3) a HPWR followed by PAA + FA.

The control for these studies was the LPWR, which involved the use of a standard garden hose to rinse each of the ten compartments of a transportation coop. A standard garden hose was moved from the left side to the right side of each compartment which took less than 30 s to perform the LPWR. Bricks were placed on one side of the coop to allow for drainage during each rinse. All treatments were given a 10 min contact time and followed by a LPWR of the transportation coops to remove any chemical residue. The concentrations that were used for all studies were the maximum concentrations recommended by the manufacturers. The HPWR used a (Briggs & Stratton, Milwaukee, WI, USA) power washer for 1 min at 3000 psi on each transportation coop.

### 2.2. Cleaners and Disinfectants

The FC that was used in specified lab trials was an alkaline/chlorine-based product (Chlor-A-Foam, Neogen Animal Safety, Lexington, KY, USA) and it was used at a 118.29 mL/L (4 oz/gal) concentration. This product contained its own foaming agent so a foam additive (FA) was not added to this product when used.

The PAA disinfectant (Peraside, Neogen Animal Safety, Lexington, KY, USA) that was used in specified trials was also used at a 118.29 mL/L (4 oz/gal) concentration. This product did not contain its own foaming agent, so a FA was added to this product when used. The FA (Perafoam, Enviro Tech Chemical Services, Inc., Modesto, CA, USA), was added at a 1% concentration.

### 2.3. Compressed Air Foam System (CAFS)

Foam is composed of air, soap, and water. We used a CAFS (Rowe CAFS LLC, Washington, AR, USA) that can produce 1874 L (495 gallons) of firefighting foam per minute. For each trial, 189.27 L (50 gallons) of tap water was measured into the tank of the CAFS followed by 5.92 L (200 oz) of FC or PAA with 1.89 L (64 oz) of the FA (PAA + FA). A 2.54 cm (1 inch) fire hose was used to apply the foam from the CAFS to the contaminated coops.

### 2.4. Transportation Coops

Four broiler chicken transportation coops (Bright Coop, Inc., Nacogdoches, TX, USA) were obtained from a local commercial broiler integrator for experimental purposes. Each coop represented an experimental unit/treatment and had ten holding compartments in a configuration of two columns with five rows. During experiments, ten pre-treatment and ten post-treatment samples were taken from each transportation coop.

The field study used three transportation coops containing market-age broilers that had recently defecated in the coops during transport to the processing plant and had probably never been cleaned.

### 2.5. Fecal Slurry

Feces were collected from single combed white Leghorn chickens housed at the Texas A&M University Poultry Research Center. Five hundred grams of organic matter, 500 mL of *Salmonella* Typhimurium (ST) and 500 mL of tap water were mixed. The ST was cultured in tryptic soy broth (Becton Dickinson and Company, Franklin Lakes, NJ, USA) for 24 h at 37 °C and passed every 8 h to be used to spike the fecal slurry before being blended and homogenized.

The final study, at the poultry processing facility, did not use the homogeneous fecal slurry method since the transport coops were recently contaminated by commercial broiler chickens. 

### 2.6. Paint Roller Application

The homogenous fecal slurry was blended and placed in a paint roller tray and a clean paint roller was used to apply the slurry onto the entrance of each compartment at a width equivalent to the length of one roller (23 cm). The slurry applied onto the transportation coops was given a 30 min dry time to simulate industry conditions.

### 2.7. Bacterial Recovery/Sampling

Samples were taken from each of the ten compartments of each transportation coop after 30 min of drying time. The samples were collected using a sterile 5 by 5 cm gauze pad which was pre-soaked with 5 mL of buffered peptone water and stored in a 4 oz WHIRL-PAK^®^ bag (NASCO, Fort Atkinson, WI, USA). A 5 by 5 cm stainless steel template was soaked in 100% ethanol and flame sterilized between samples. To avoid sampling overlap, all pre-treatment samples were taken from the left side of each compartment, and all post-treatment samples were taken from the right.

### 2.8. Culture

Samples were kept in 4 oz WHIRL-PAK^®^ bags and homogenized by a stomacher blender (Seward Limited, Worthing, West Sussex, United Kingdom for 30 s at 265 rpm. A series of 10-fold dilutions were performed into Butterfield’s dilution tubes, plated onto tryptic soy agar (Becton Dickinson and Company, Franklin Lakes, NJ, USA) and incubated for 24 h at 37 °C.

For lab trials 1 and 2 the addition of Xylose-Lysine-Tergitol 4 (XLT4; Becton Dickinson and Company, Franklin Lakes, NJ, USA) plates were used to evaluate Salmonella bacterial recovery and were plated from the same Butterfield’s dilution tubes then incubated for 48 h at 37 °C. Sample WHIRL-PAK^®^ bags were incubated for 24 h at 37 °C then 100 μL of each sample were transferred into corresponding Rappaport-Vassiliadis (RV) Salmonella enrichment broth(Becton Dickinson and Company, Franklin Lakes, NJ, USA). The RV tubes that were incubated for 24 h at 37 °C were then struck onto XLT4 plates and incubated for 24 h at 37 °C to determine how many positive samples were detected through selective enrichment.

### 2.9. Scanning Electron Microscopy

Transport coop flooring was cut into approximately 18 mm squares and then thoroughly washed and cleaned in 100% ethanol. Squares were then packaged and sterilized by ethylene oxide gas sterilization. Sterilized squares were individually placed in 50 mL conical centrifuge tubes containing 45 mL of tryptic soy both inoculated with 10 µL from an overnight culture of *Enterococcus faecalis* and incubated overnight at 37 °C on a horizontal shaker. After overnight incubation, squares were removed and preserved by emersion in 25 mL of a fixative containing 3% glutaraldehyde prepared in 50 mM phosphate buffer, pH 7.4, with 50 mM sucrose. After a 60 min incubation, squares were post-fixed in a solution of 1% osmium tetroxide in 100 mM phosphate buffer, pH 7.4, with 100 mM sucrose for an additional 60 min. Following osmication, squares were rinsed in distilled water, dehydrated in a graded ethanol series and critical point dried using CO_2_. Squares were then mounted on aluminum stubs, sputter-coated with gold and examined using a scanning electron microscope (JEOL 6400, JEOL USA, INC, Peabody, MA, USA). Control squares were un-inoculated pieces of cage material that were placed on aluminum stubs and examined in the scanning electron microscope.

### 2.10. Statistical Analysis

Bacterial recovery data were subjected to a one-way ANOVA using the general linear model procedure (SAS Institute Inc., Cary, NC, USA), with means deemed significantly different at *p* < 0.05 and separated using Duncan’s multiple range test.

## 3. Results and Discussion

The objective for lab trial 1 was to spike layer feces with *Salmonella* Typhimurium and evaluate whether a high-pressure water rinse (HPWR) step prior to or following the PAA + FA treatment would be an added benefit in reducing aerobic bacteria and Salmonella (Table 1). Transportation coops treated with PAA + FA alone or with a HPWR step prior to or following the treatment in both replications were statistically similar (*p* < 0.05) in reducing aerobic bacteria (4.10 to 5.17 logs) and Salmonella (3.99 to 4.58). The LPWR consistently had the lowest reductions in both replications when reducing aerobic bacteria (2.09 and 2.14 logs) and Salmonella (2.10 and 2.16 logs).

The objective for lab trial 2 was to spike the feces with *Salmonella* Typhimurium and evaluate whether a HPWR step prior to or following a FC would be an added benefit in reducing aerobic bacteria and Salmonella (Table 2). Treatments using a FC varied statistically in both replications. In replication 1, HPWR prior to the FC and the FC used alone had the greatest reductions and were statistically similar (*p* < 0.05) in reducing aerobic bacteria (4.05 and 4.23 logs, respectively). The FC followed by the HPWR was statistically different (*p* < 0.05) from all other treatments at 3.5 log_10_ reductions of aerobic bacteria and was greater than the LPWR. The LPWR had the lowest reduction of aerobic bacteria at 1.12 logs.

In the same lab trial *Salmonella* Typhimurium recovery was also evaluated and all three treatments using the FC were statistically similar (*p* < 0.05) to one another (3.17 to 3.65 logs). The LPWR had the lowest reduction at 1.82 logs of Salmonella and was statistically different than all other treatments. These data demonstrate that the FC is effective in reducing not only aerobes but Salmonella as well. 

In replication 2 of lab trial 2 (Table 2) aerobic bacteria reductions for all coops were statistically different from one another. The greatest reduction was achieved from the HPWR followed by the FC, which was a 4.84 log_10_ reduction of aerobic bacteria. Another significant reduction came from the FC used alone with a reduction at 3.59 logs of aerobes. The FC followed by the HPWR with a reduction of 2.78 logs of aerobic bacteria also had a significant reduction. The lowest reduction was observed with the LPWR treatment at 0.98 log reduction of aerobic bacteria.

Replication 2 also evaluated the reductions of Salmonella. The authors found that the HPWR followed by the FC had the greatest statistically significant reduction of 3.90 logs of aerobic bacteria. The HPWR used prior to the use of the FC consistently proved to be the most effective way to reduce aerobic bacteria and Salmonella in both replications, which could be because the organic matter was removed prior to the product being applied. The organic matter that was used for lab trials had water added and *Salmonella* Typhimurium was blended to allow the slurry to be thicker in consistency and truer to organic matter that is naturally present on broiler transportation coops. According to Dvorak and Peterson 2009 [18] the removal of organic matter first is essential because it acts as a barrier to the microorganisms present and affects the efficacy of the disinfectant. They concluded that the efficacy of hypochlorites is rapidly reduced when a large amount of organic matter is present. Perhaps this is why we saw better results from the coops treated by the HPWR first followed by the disinfectant or cleaner in this lab trial. The FC used alone and followed by the HPWR statistically had similar reductions (2.82 and 3.18 logs). Finally, the LPWR statistically showed that it had the lowest reductions of Salmonella at 0.65 logs of aerobic bacteria.

The objective of the field trial was to evaluate whether PAA + FA alone or after a HPWR step would be effective in reducing aerobic bacteria on freshly contaminated broiler transportation coops from a poultry processing facility (Table 3). Similar results were seen in both replications. Significant reductions (1.72 and 2.32 logs, respectively) of aerobic bacteria were observed from coops treated with HPWR followed by PAA + FA in both replications. The HPWR proved to be effective in a field setting, which may be due to the removal of organic matter present that had not been washed previously.

Researchers have suggested that high-pressure rinsing may be more effective to significantly reduce bacterial load than a LPWR and was what was seen in the results for our lab trials [3]. Their hypothesis to apply a HPWR proved to be effective in a field setting along with removal of organic matter which is what previous research and literature suggests. Stringfellow and co-workers [19] concluded that when using disinfectants, correct contact time, temperature, and amount of organic matter affects product efficacy. Furthermore, a multi-step protocol is required to effectively reduce the bacterial load found in cages [20]. The higher amount of organic matter seen in the present study led to the conclusion that the addition of a HPWR will further reduce bacterial load present on transportation coops. The PAA + FA used alone had a significant reduction of aerobic bacteria (0.88 and 0.80 logs). The LPWR had the lowest reduction concentrations (0.0 and 0.42 logs) for the field trials conducted.

**Figure 1:**
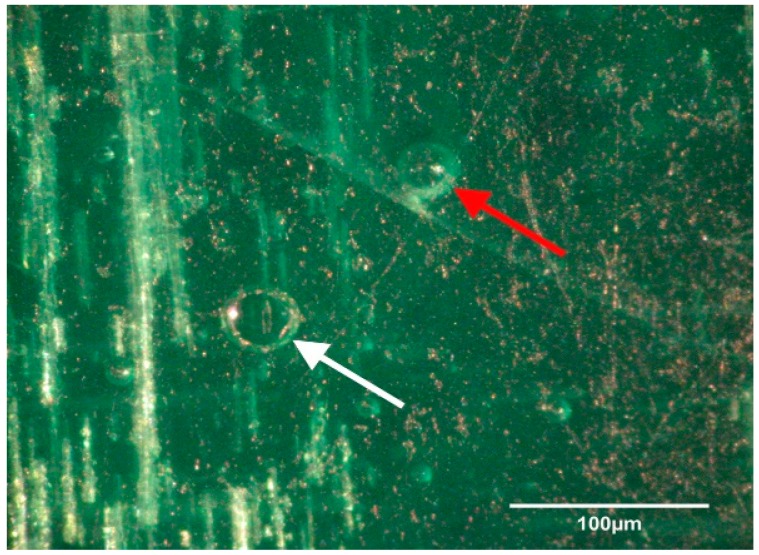
Light micrograph of uninoculated fiberglass flooring material depicting a hole (white arrow) in the material as well as subsurface air bubbles (red arrow). Sub-surface bubbles can be exposed to surface contamination as the surface wears with age.

**Figure 2:**
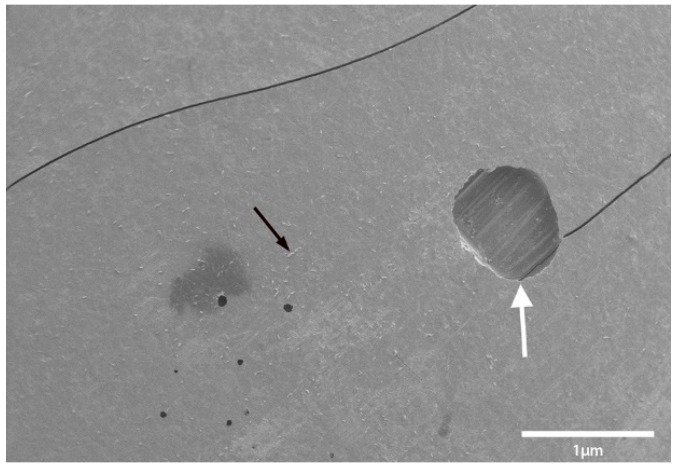
White arrow indicates hole in the surface of the fiberglass floor like that shown in Figure 1 (white arrow). Black arrow indicates bacteria colonizing the surface of the floor.

**Figure 3:**
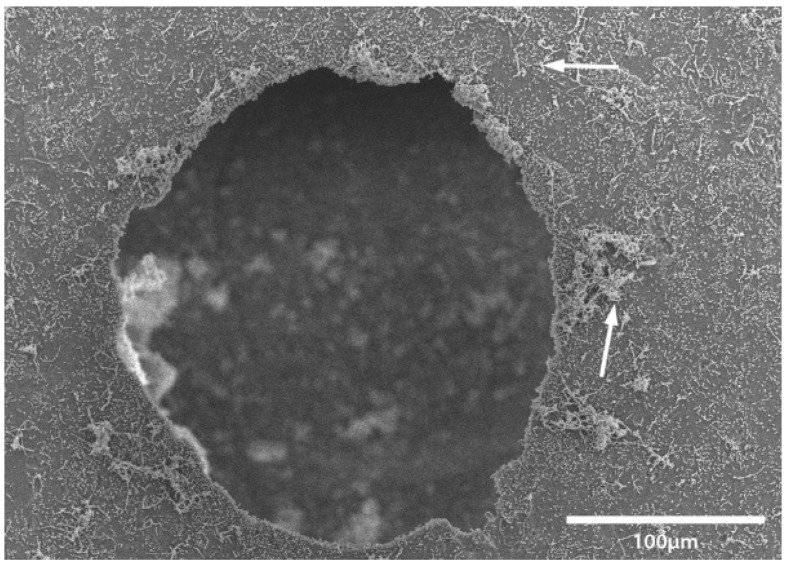
Higher magnification of a hole as seen in Figure 2 at 72 hours post inoculation with bacterial aggregates (white arrows).

**Figure 4:**
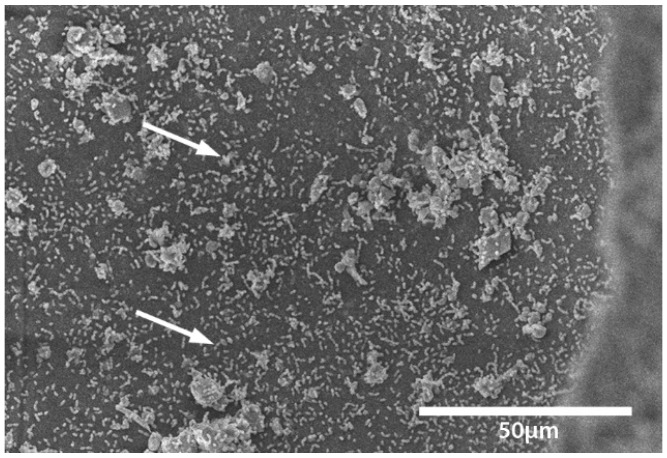
Micrograph of the bottom of the hole seen in Figure 3. Large aggregates of bacteria are evident adhering to the area of the hole (white arrows).Figure 2, Figure 3 and Figure 4: Scanning electron micrographs of bacteria inoculated flooring at various timepoints after inoculation and magnifications.

Transport coop floors were power washed and treated with relatively high concentrations of disinfectant or cleaner during the lab and field trials. The researchers were surprised to continue to find bacteria present on surfaces that appeared to be smooth and clean. To further investigate this observation, a separate experiment was conducted within a microbiology lab. A coupon of broiler transport coop flooring was inoculated with *Enterococcus faecalis* and evaluated by a light and electron microscope (Figure 1, Figure 2, Figure 3 and Figure 4). We found that the apparent smooth surfaces were actually scratched and covered in pits where bacteria could accumulate. It is possible that microbes may never encounter a cleaner or disinfectant due to the protection provided due to these imperfections. 

The current study did not demonstrate whether the bacteria present were killed on the coops or were physically washed away but shows whether the bacteria were reduced or removed. As such, this observation may be irrelevant since the bacteria are no longer present on the transportation coops that can be a vehicle for cross-contamination. Continued research in a commercial setting may be needed. Furthermore, evaluations of bacterial counts on carcasses can be compared when taken from washed transport coops versus unwashed coops to further determine bacterial load. These products have already been approved by the Environmental Protection Agency, which means that they may be implemented in being used at a poultry processing facility. These data suggest that a CAFS application of cleaners and disinfectants may be used to significantly reduce Salmonella and aerobic bacteria on broiler transport coops. While a direct comparison was not made, coops from a commercial setting were found to be more difficult to clean and disinfect than coops which were contaminated in the laboratory.

## 4. Conclusions

A CAFS can be used to apply disinfectants with foam or foaming cleaners to effectively reduce aerobic bacteria which contaminate broiler transportation coops.

Common poultry disinfectants (peroxyacetic acid and chlorine releasing agents) when combined with a foam additive prove to be effective in reducing aerobic bacteria and Salmonella.

The addition of a HPWR used prior to or following the treatment did not improve efficacy in a laboratory setting but was beneficial in the field study.

## Figures and Tables

**Table 1:** Lab Trial 1: Reduction of aerobic bacteria and Salmonella on broiler transportation coops following a compressed air foam application of disinfectant and a high-pressure water rinse.^1.^

Treatment ^2^	Rep1 Log_10_ Reductions Aerobic Plate Count ^3,4^	Log_10_ Reductions Salmonella Plate Count	Direct Plating Incidence ^5^	Selective Enrichment Incidence	Rep2 Log_10_ Reductions Aerobic Plate Count	Log_10_ Peductions Salmonella Plate Count	Direct Plating Incidence	Selective Enrichment Incidence
**LPWR**	2.14 *^,b^ ± 0.47	2.10 ^b^ ± 0.54	10/10	10/10	2.09 ^b^ ± 0.29	2.16 ^b^ ± 0.38	10/10	10/10
**PAA + FA Only**	4.71 ^a^ ± 1.33	4.12 ^a^ ± 0.26	1/10	10/10	4.77 ^a^ ± 1.17	4.22 ^a^ ± 0.41	3/10	10/10
**HPWR followed by PAA + FA**	4.10 ^a^ ± 0.81	3.99 ^a^ ± 0.65	1/10	9/10	5.17 ^a^ ± 0.93	4.48 ^a^ ± 1.03	0/10	8/10
**PAA + FA followed by HPWR**	4.42 ^a^ ± 1.38	4.58 ^a^ ± 1.21	1/10	5/10	4.89 ^a^ ± 1.34	4.35 ^a^ ± 1.35	2/10	7/10

^1^ All treatments were given a 10 min contact time and were followed by a LPWR of the transportation coops to remove any residual chemical. ^2^ LPWR = Low-pressure water rinse; PAA + FA = Peroxyacetic acid with a foam additive, and HPWR = High-pressure water rinse. ^3^ Values for reductions in aerobic bacteria and Salmonella recovery were calculated by subtracting post-treatment from pre-treatment samples. ^4^ Data are presented as mean ± SE, log_10_ reduction; *n* = 10 pooled samples per treatment; log reductions are subjected to a one-way ANOVA using the GLM procedure, with means deemed significantly different at *p* < 0.05 and separated using Duncan’s multiple range test.^5^ Salmonella incidence data is described as “X” out of 10 samples or 10/10 for 100% positive samples. * ^a-b^ Column values with different superscripts differ significantly (*p* < 0.05).

**Table 2:** Lab Trial 2: Reduction of aerobic bacteria and Salmonella on broiler transportation coops following a compressed air foam application of foam cleaner and a high-pressure water rinse.^1.^

Treatment ^2^	Rep1 Log_10_ Reductions Aerobic Plate Count ^3,4^	Log_10_ Reductions Salmonella Plate Count	Direct Plating Incidence ^5^	Selective Enrichment Incidence	Rep 2 Log_10_ Reductions Aerobic Plate Count	Log_10_ Reductions Salmonella Plate Count	Direct Plating Incidence	Selective Enrichment Incidence
**LPWR**	1.12 *^,c^ ± 0.39	1.82 ^b^ ± 0.46	10/10	10/10	0.98 ^d^ ± 0.51	0.65 ^c^ ± 0.95	10/10	10/10
**FC Only**	4.05 ^a^ ± 0.71	3.71 ^a^ ± 0.59	3/10	10/10	3.59 ^b^ ± 0.81	3.18 ^b^ ± 0.85	5/10	10/10
**HPWR followed by FC**	4.23 ^a^ ± 0.53	3.48 ^a^ ± 0.54	2/10	10/10	4.84 ^a^ ± 1.05	3.90 ^a^ ± 0.33	1/10	10/10
**FC followed by HPWR**	3.50 ^b^ ± 0.13	3.65 ^a^ ± 0.13	0/10	10/10	2.78 ^c^ ± 0.74	2.82 ^b^ ± 0.72	8/10	10/10

^1^ All treatments were given a 10 min contact time and were followed by a LPWR of the transportation coops to remove any residual chemical. ^2^ LPWR = Low-pressure water rinse; FC = Foam cleaner; and HPWR = High-pressure water rinse. ^3^ Values for reductions in aerobic bacteria and Salmonella recovery were calculated by subtracting post-treatment from pre-treatment samples. ^4^ Data are presented as mean ± SE, log_10_ reduction; *n* = 10 pooled samples per treatment; log reductions are subjected to a one-way ANOVA using the GLM procedure, with means deemed significantly different at *p* < 0.05 and separated using Duncan’s multiple range test.^5^ Salmonella incidence data is described as “X” out of 10 samples or 10/10 for 100% positive samples. *^, a-b^ Column values with different superscripts differ significantly (*p* < 0.05).

**Table 3:** Field Trial—Reduction of aerobic on broiler transportation coops following a compressed air foam application of disinfectant and a high-pressure water rinse.^1.^

Treatment ^2^	Replication 1 Log_10_ Reductions Aerobic Plate Count	Replication 2 Log_10_ Reductions Aerobic PlateCount
**LPWR**	0.00 *^,c^ ± 0.66	0.42 ^c^ ± 0.37
**PAA + FA**	0.88 ^b^ ± 0.62	0.80 ^b^ ± 0.34
**HPWR followed by PAA + FA**	1.72 ^a^ ± 0.57	2.32 ^a^ ± 0.40

^1^ All treatments were given a 10 min contact time and were followed by a LPWR of the transportation coops to remove any residual chemical. ^2^ LPWR = Low-pressure water rinse; PAA + FA = Peroxyacetic acid with a foam additive, and HPWR = High-pressure water rinse. ^3^ Values for reductions in aerobic bacteria were calculated by subtracting post-treatment from pre-treatment samples. ^4^ Data are presented as mean ± SE, log_10_ reduction; *n* = 10 pooled samples per treatment; log reductions are subjected to a one-way ANOVA using the GLM procedure, with means deemed significantly different at *p* < 0.05 and separated using Duncan’s multiple range test. *^, a-b^ Column values with different superscripts differ significantly (*p* < 0.05).
